# Learning and STEM identity gains from an online module on sequencing-based surveillance of antimicrobial resistance in the environment: An analysis of the PARE-Seq curriculum

**DOI:** 10.1371/journal.pone.0282412

**Published:** 2023-03-10

**Authors:** Scarlet S. Bliss, Eve A. Abraha, Erica R. Fuhrmeister, Amy J. Pickering, Carol A. Bascom-Slack

**Affiliations:** 1 Levy Center for Integrated Management of Antimicrobial Resistance, Tufts University, Medford, Massachusetts, United States of America; 2 Center for Science Education, Department of Medical Education, Tufts University School of Medicine, Boston, Massachusetts, United States of America; University of Mississippi, UNITED STATES

## Abstract

COVID-19 necessitated the rapid transition to online learning, challenging the ability of Science, Technology, Engineering, and Math (STEM) professors to offer laboratory experiences to their students. As a result, many instructors sought online alternatives. In addition, recent literature supports the capacity of online curricula to empower students of historically underrepresented identities in STEM fields. Here, we present PARE-Seq, a virtual bioinformatics activity highlighting approaches to antimicrobial resistance (AMR) research. Following curricular development and assessment tool validation, pre- and post-assessments of 101 undergraduates from 4 institutions revealed that students experienced both significant learning gains and increases in STEM identity, but with small effect sizes. Learning gains were marginally modified by gender, race/ethnicity, and number of extracurricular work hours per week. Students with more extracurricular work hours had significantly lower increase in STEM identity score after course completion. Female-identifying students saw greater learning gains than male-identifying, and though not statistically significant, students identifying as an underrepresented minority reported larger increases in STEM identity score. These findings demonstrate that even short course-based interventions have potential to yield learning gains and improve STEM identity. Online curricula like PARE-Seq can equip STEM instructors to utilize research-driven resources that improve outcomes for all students, but support must be prioritized for students working outside of school.

## Introduction

A research or laboratory experience often enhances an undergraduate education in the science, technology, mathematics, and engineering (STEM) fields [1–4]. Significant research and resources have been channeled into the creation, dissemination and evaluation of course-based undergraduate research experiences (CUREs). CUREs are a mechanism to increase educational inclusivity by removing barriers that accompany out-of-class and/or unpaid research experiences [5–8] and they have been shown to increase students’ STEM identity and sense of belonging [9–11] as well as other positive outcomes, including high levels of ownership, discovery, iteration, and confidence in career intentions [12–15].

### Pedagogical motivations: COVID-19, STEM identity

Before the COVID-19 pandemic, the Prevalence of Antibiotic Resistance in the Environment (PARE) Project was implemented in university and community college settings nationally as a series of short course-based laboratory modules designed to expose students to fundamental biology research while allowing them to participate in work combatting the emerging public health concern of antimicrobial resistance (AMR). The modules are focused on culture- and molecular-based wet lab approaches using soil samples for environmental surveillance of AMR [16].

COVID-19 necessitated the rapid transition of schools to remote and online learning, which challenged the ability of STEM professors to offer laboratory research experiences to their students). Over 94% of learners globally were impacted, necessitating both learning and research experiences to be converted to digital formats [17, 18]. To help fill this identified gap in student opportunity, our research team developed PARE-Seq, an online, open-source short module teaching bioinformatics methods for environmental surveillance of antibiotic resistance research. PARE-Seq is an extension of the original PARE curriculum.

Beyond the need to adapt to a virtual learning and research environment, we saw development of an online curriculum for undergraduates as an opportunity to integrate mechanisms to empower students of historically underrepresented identities in STEM fields and explore how these pedagogical decisions could impact student STEM identity [1, 19, 20]. In the U.S., marginalized communities comprise a significantly lower portion of jobs in STEM fields than the overall workforce. Hispanic/Latine workers make up 17% of the workforce but only 8% of STEM workers; Black workers comprise 11% of total employment in the U.S but 9% of STEM workers [21]. Women make up 50% of the STEM workforce, but a higher percentage are in health-related jobs (74%), and only 15% of engineers are female-identifying [21].

Science identity (or STEM identity), broadly defined, is the aspect of self that relates to science [22]. Research on STEM identity has predominantly relied on a qualitative approach, which was necessary to define and provide a rich understanding of the concept [22, 23]. However, we sought to conduct a quantitative assessment of changes in STEM identity pre- and post-participation, through use of a previonsly validated single-item instrument [24]. We chose to build a module with an undergraduate, female, and racially diverse teaching team, with the aim of providing faces relatable to underrepresented identities in the field.

### Content motivations: Bioinformatics, antimicrobial resistance

Added to the pedagogical motivations of PARE-Seq, we saw an opportunity to expose undergraduates to DNA sequencing data and bioinformatics, a rapidly-growing interdisciplinary field spanning computer science and biology [25]. Providing opportunities for students to learn some of the most powerful approaches used in the field can result in advantages when entering the workforce [26–29]. Bioinformatics provides easy and cost-effective opportunity for students to practice the iterative process of science, a critical component of classroom research [13], making bioinformatics advantageous to the CURE format [25, 27, 30]. Bioinformatics research also allows undergraduates to practice trial and error, what Lopatto et al., 2020 recognized as ‘formative frustration’, an integral element of the scientific process.

Leveraging modern molecular approaches, such as long-read sequencing, also allows us to understand complex environmental transmission routes, a second content motivation for PARE-Seq [31, 32]. Antimicrobial resistance (AMR) is a growing threat to human health in the United States and globally. Recent predictions estimate 4.95 million deaths worldwide were associated with bacterial AMR in 2019 [33]. Trends show that the prevalence of AMR is rising in many common pathogens, such as *Escherichia coli*, *Klebsiella pneumoniae*, and *Staphylococcus aureus*, and by 2050, an estimated 10 million deaths per year could result from AMR [33–35]. With subject-area expertise in AMR among our research group and seeing an opportunity to involve undergraduates in this research, AMR was the second content motivation of PARE-Seq development.

The purpose of this study is to analyze the effectiveness of the PARE-Seq module that seeks to educate students on molecular and bioinformatics approaches to AMR research and environmental surveillance methods in public health. We add to existing knowledge on bioinformatics pedagogy and undergraduate research experiences by answering the questions of (a) whether this short online module can effectively teach students about molecular methods and computational aspects of environmental surveillance work, (b) if it is an impactful remote research experience for diverse student audiences, and (c) if the module might influence students’ identity in regards to STEM.

### Research objectives

Our study took place during Spring 2021 with PARE-Seq embedded within Biology, Genetics, Molecular Biology and Microbiology courses at a range of undergraduate institutions. Most of these students were continuing to learn this material remotely due to the COVID-19 pandemic. Using the pre- and post-surveys iterated on during a Fall 2020 pilot of PARE-Seq, we asked the following three research questions (RQs):

1. Did students experience significant learning gains from participating in the PARE-Seq curriculum?
2. Did student experience significant changes in STEM identity from participating in PARE-Seq?
3. Were there differential learning gains for students based on sociodemographic characteristics?
4. Were there differential changes in STEM identity based on sociodemographic characteristics?

## Methods

### Curriculum development and revision

**Fig 1:**
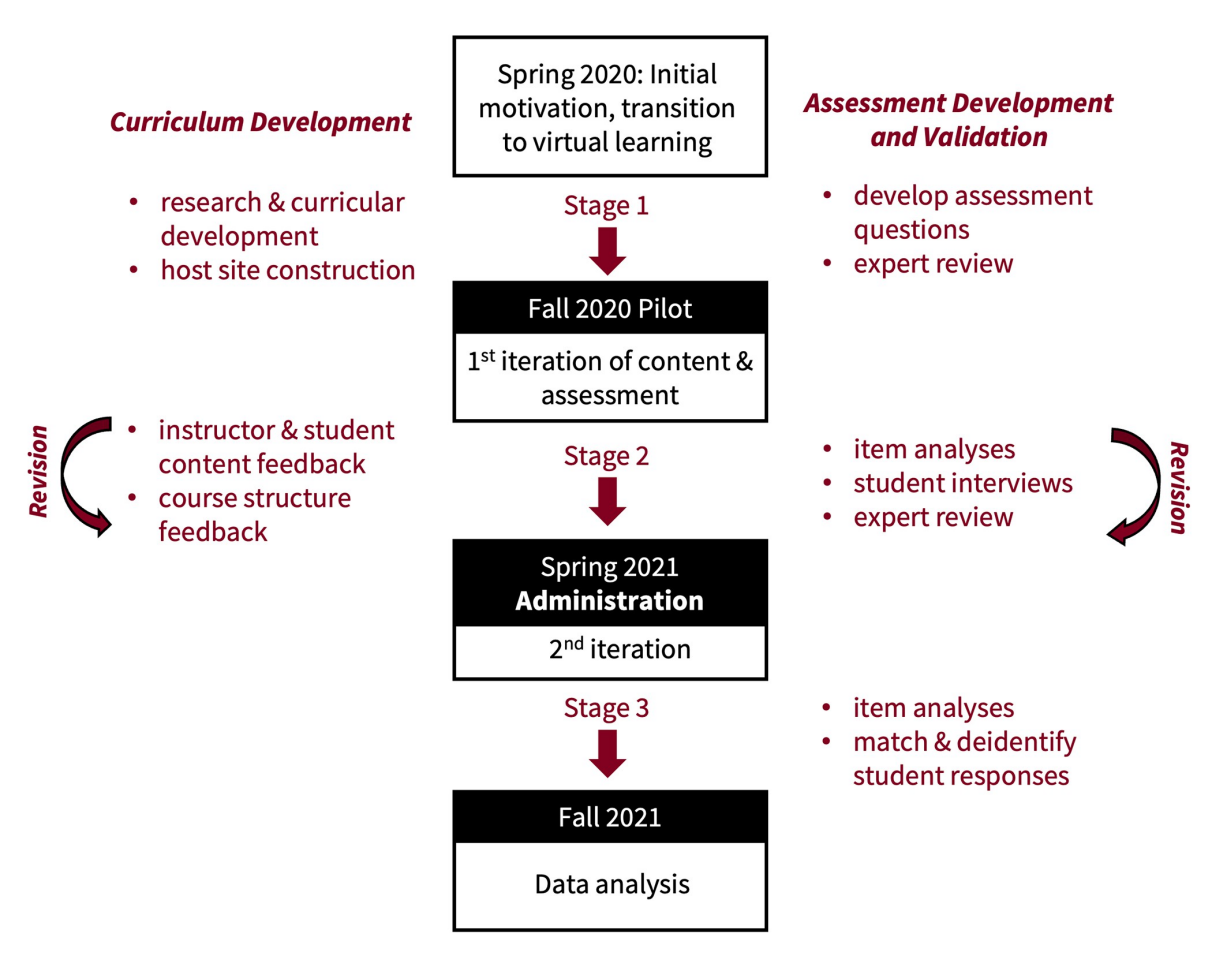
PARE-Seq curriculum and assessment tool development and revision process. Both curriculum and assessment instrument were revised between the Fall 2020 pilot and Spring 2021 administration.

Development of the curriculum was a multi-stage process involving curricular content development, choosing a bioinformatics platform, bioinformatics pipeline development, course video production, and host web platform development (Fig 1). The format was modeled from substantive exploration of virtual learning platforms for STEM, including Coursera, LinkedIn Learning, and Canvas, as well as discussions with bioinformaticians and professors at Tufts University. We also developed an instructor manual and hosted instructional webinars for participating faculty.

PARE-Seq learning objectives, as well as the course content and assignments, were designed through a pilot and administration phase (Fig 1). Following the first iteration of the course in Fall 2020 with 12 institutions, we conducted instructor and student feedback surveys to revise both course content and structure for Spring 2021 administration (see S1 and S2 Files). Based on feedback, we clarified content in the lecture videos and added subtitles as well as lecture transcripts to the course site to improve accessibility.

### Assessment development and revision

The goal of the pre- and post- assessment was to evaluate students’ learning from the bioinformatics activity (Parts 2 and 3). We began development by creating a set of questions aligned to our learning objectives. We recruited instructors at 12 institutions to pilot PARE-Seq in fall of 2020 and launched the first iteration of the module and assessment with the Fall 2020 cohort of students.

Next, we conducted item analyses on the pilot assessment data. Difficulty and discrimination indices identified the proportion of students answering the question correctly and the question’s ability to distinguish between high-performing and low-performing students [36]. A point-biserial correlation measures question reliability by comparing student performance on individual questions with their total scores. Values range from -1.0 to +1.0 and will be positive if students with higher total scores are more likely to answer the question correctly. Additionally, Cronbach’s alpha was calculated using R 4.1.2 statistics software. This value was a measure of internal reliability of the survey as a whole.

Based on statistical findings from the item analysis, questions that performed poorly were removed or modified, and new questions were added. The new draft multiple-choice assessment was then reviewed by four faculty experts in bioinformatics or molecular biology at Tufts University. We carried out student cognitive interviews on the updated assessment, asking individuals to explain their response choices for each question in depth [37]. Generally, students noted that any questions asking them to apply their training from the bioinformatics activity were not interpretable in the pre-assessment, but upon completion of the course they could demonstrate understanding of the concepts.

The final pre- and post-assessment was a ten-item MC and T/F tool that was higher performing on item analyses, suggesting it more accurately assessed student learning gains in Spring 2021 compared to Fall 2020. Please see *Results* for details.

### Study participants

This study was conducted between January 2021 and May 2021 at a range of public and private universities, community colleges, and high schools nationwide. Institutions were recruited based on prior participation in the PARE curricula. Courses in which the module was taught included Introductory Biology, Microbiology, Genetics, and Biology elective courses. A total of 176 students completed the pre-assessment and 165 completed the post-assessment. Participants were excluded if they could not be matched pre- to post-intervention, i.e., they did not take both surveys. Removal also occurred if the participant did not complete all the sociodemographic questions in the post-survey, or if they did not consent to participate in the study. After removing these records plus responses from international students and those under 18, our final study sample contained 101 records. 83% attended doctoral or professional degree granting universities and 17% attended associates or community colleges, according to Carnegie basic classification. Student host institutions were labeled as School A—D for participant confidentiality.

79.2% of students were female and 20.8% were male (a range of gender identities were offered in the survey, but all students identified as male or female). Sociodemographic characteristics of participating students are indicated in Table 1. Students completed the assessment in an online survey via Qualtrics outside of class time. To incentivize student participation, instructors were encouraged to give students a small amount of regular or extra credit for the assignment, with the exact amount being at the discretion of the instructor. This study was approved by the Tufts Social & Behavioral Institutional Review Board (#00000962), and written consent was obtained via the Qualtrics survey (see S3 File). Only data from those students who gave their informed consent were included in this study.

**Table 1:** Student self-reported sociodemographic characteristics.

Sociodemographic characteristic	*n (%)* ^*a*^
Institution (Carnegie basic classification)^b^	
*A (Doctoral/Professional University)*	49 (48.5%)
*B (Doctoral/Professional University)*	35 (34.7%)
*C (Baccalaureate/Associate’s College*: *Associate’s Dominant)*	10 (9.9%)
*D (Associate’s College)*	7 (6.9%)
Gender	
*Male*	21(20.8%)
*Female*	80 (79.2%)
Race/ethnicity	
*White*	64 (63.4%)
*Black/African American*	16 (15.8%
*Hispanic/Latine*	9 (8.9%)
*Asian/Asian-American*	4 (4.0%)
*Middle Eastern/North African*	1 (1.0%)
*Biracial/Multiracial*	6 (5.9%)
Parental education	
*4-year degree or more*	59 (58.4%)
*No parent graduated college*	39 (38.6%)
Extracurricular work hours	
*No extracurricular work hours*	42 (41.6%)
*0–10 hours*	14 (13.9%)
*11–20 hours*	22 (21.8%)
*21–30 hours*	10 (9.9%)
*31+ hours*	12 (11.9%)
Self-rated quality of workspace (1–6 rating scale)	
*Few distractions/good quality (4–6)*	69 (68.3%)
*Many distractions/poor quality (1–3)*	32 (31.7%)
*Total*	101

^a^Numbers may not sum to 101 due to missing data.

^b^Institution names omitted for participant anonymity.

### Statistical analysis

**Fig 2:**
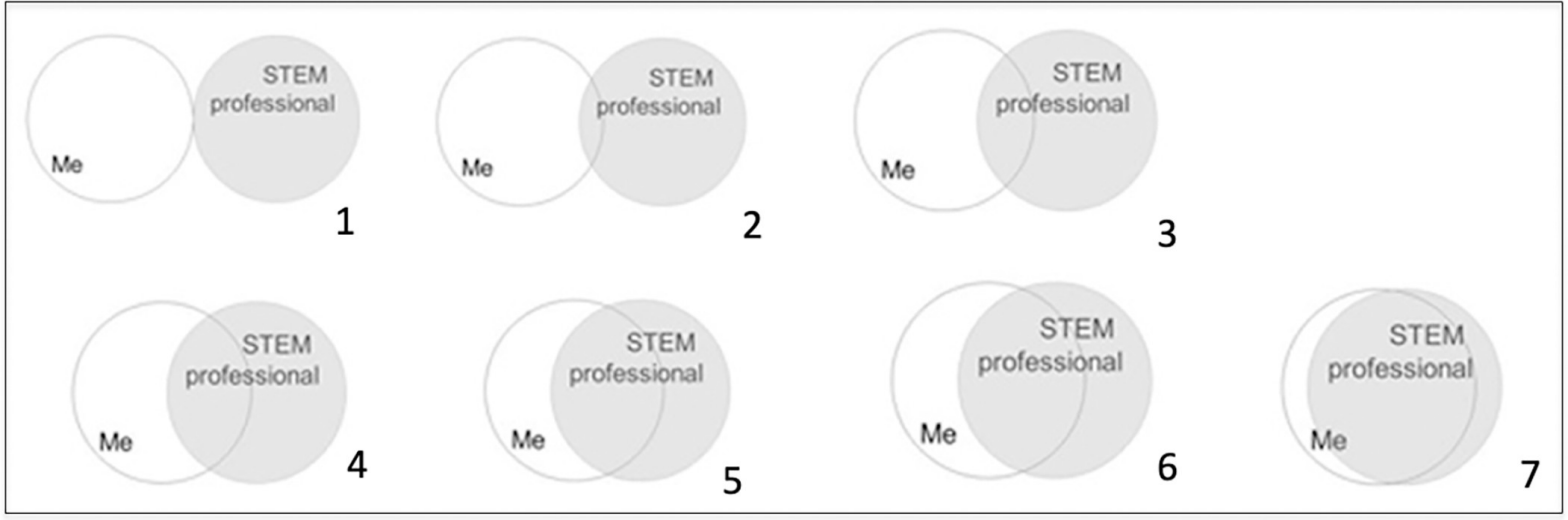
Single item measure of STEM identity used in PARE-Seq surveys. Students were asked: Please select the picture above that best describes the current overlap of how you see yourself and what your image of a STEM professional is (McDonald et al., 2019).

Primary outcomes of the following analysis included 1) a learning gains score equaling the difference between pre- and post-assessment (numeric, between -10 and +10, henceforth referred to as learning gains), and 2) change in STEM identity score from pre- to post-intervention (numeric, between -6 and 6, referred to as STEM identity score). To answer RQ2, we employed a previously validated, multiple-choice question developed by McDonald et al. (2019) to assess student STEM Identity in the sciences from pre- to post-intervention (Fig 2). To answer RQs 3 and 4, we asked a set of sociodemographic questions following the post-assessment to gather information on student self-identified gender, race/ethnicity, parental education, hours worked per week outside of school, self-rated quality of home workspace, and access to technology.

We used R software (version 4.1.2) to conduct all statistical analyses. We employed the “effsize” and “ggplot2” for effect size calculations and figures, respectively [38, 39]. Prior to answering the RQs, we performed a series of item analyses on the Fall 2020 pilot survey and the updated Spring 2021 ten-item multiple choice assessment, including index of difficulty, item discrimination index, coefficient alpha and point-biserial correlation to assess the difficulty, reliability, and discriminatory power of the content questions in the survey.

Paired t-tests and effect size estimates were conducted to assess learning gains and change in STEM identity score. For RQs 3 and 4 we constructed multivariate regression models. Predictor variables for each outcome were included based on the questions asked in the post-assessment, hypothesized a priori to explain variance in students’ learning gains and change in STEM identity score. Residuals plots and multicollinearity tests were assessed to ensure no non-linear trends or correlation between predictors.

For both outcomes, we explored the effects of student gender, race/ethnicity, host institution, parent education level, extracurricular work hours per week, level of completion of the PARE-Seq, and self-rated quality of students’ workspace. We first examined effects of covariates by bivariate analyses and subsequently report final models of learning gains and change in STEM identity score. No further model selection was conducted.

## Results

### Curriculum design

**Fig 3:**
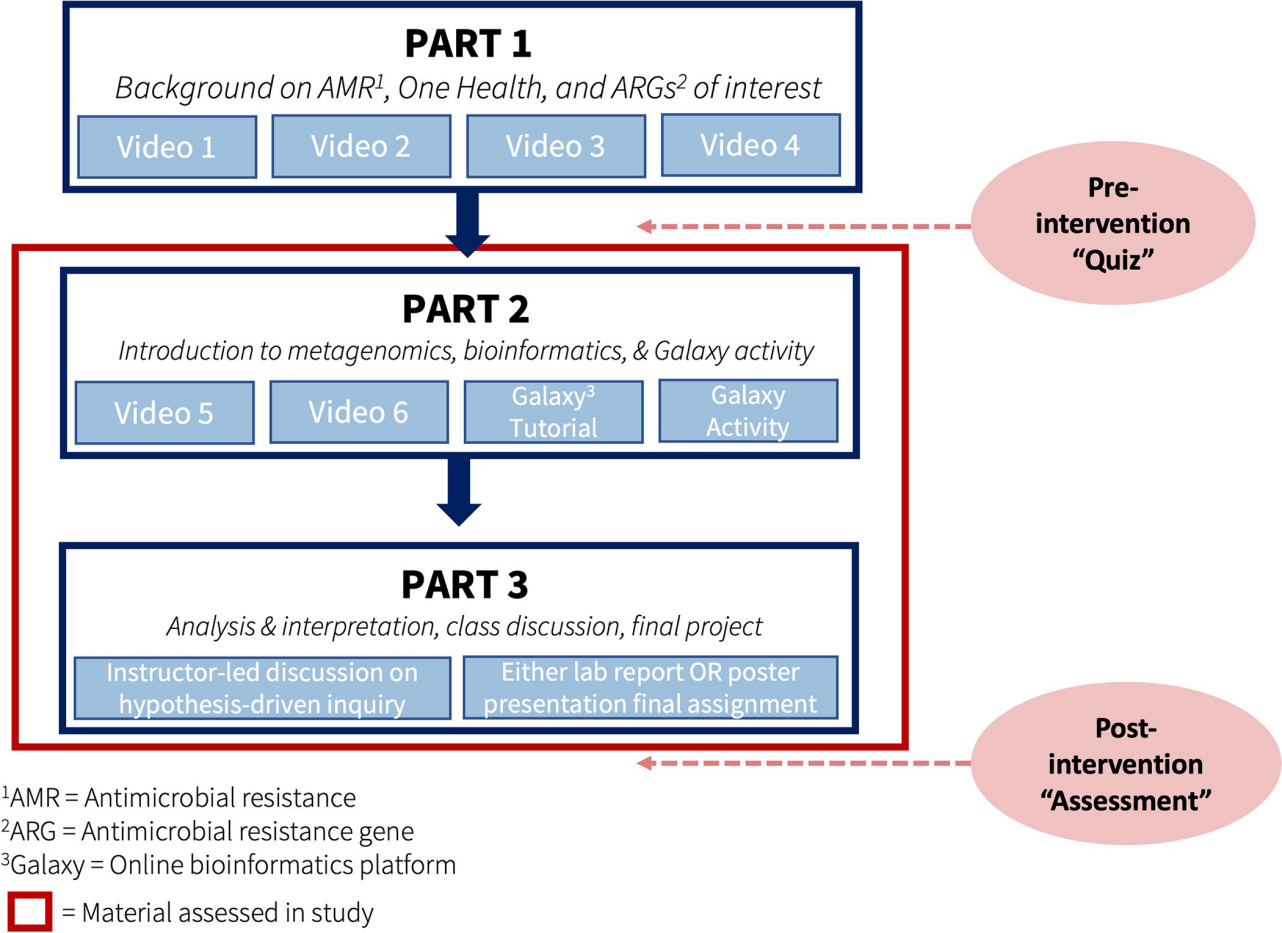
PARE-Seq curriculum and administration timepoints of pre- and post-assessment. PARE-Seq was designed to be administered as a module within an undergraduate or advanced high school biology course.

With the onset of the pandemic, faculty teaching the Prevalence of Antibiotic Resistance in the Environment CURE needed to quickly identify a virtual activity that could substitute for this wet-lab based activity. Over the course of five months, we developed a three-part suite of materials to assist instructors during this challenging time (Fig 3). Part 1 consists of four 10 to 20 minute videos to provide basic background in antimicrobial resistance related, but not essential to the bioinformatics activity. Part 2 is a bioinformatics activity that can be completed in approximately one hour. Part 3 is an instructor-guided discussion and analysis of the bioinformatics results plus instructions for two alternate student assignments—a poster or lab report.

We designed the instructional videos (for Parts 1 and 2) to uniquely center one white and one Black-identifying female undergraduate student as the course instructors, as substantive research has indicated that representation by minority gender and racial identities in STEM may support learning by those underrepresented student demographics [22, 40–43]. For the bioinformatics analysis activity (Part 2), we chose Galaxy, an open-source, web-based tool to create and use bioinformatics workflows. Students can access Galaxy and complete the analysis from home on their personal computers. In our workflow, students analyze actual soil and water samples from India and Kenya (obtained through prior research studies by the Pickering Lab). The bioinformatics workflow was designed to identify antimicrobial resistance genes present in the samples. To conclude the project (Part 3), after instructor-led discussion, students reported their findings through either a lab report or poster presentation (Fig 3).

We defined 7 learning objectives for PARE-Seq, informed by Bloom’s taxonomy for learning, teaching, and assessing [44]. The videos and activities in Parts 2 and 3 directly related to one or more of the objectives, and they were outlined for students in the introduction video. Table 2 lists the final set of learning objectives and the corresponding items from the pre- and post-assessment that relate.

**Table 2:** PARE-Seq learning objectives and associated pre- and post- assessment questions.

Learning Objective	Associated Questions^1^
*1*. *Explain* the major advantage/disadvantage of using a sequence-based approach vs. PCR-based detection of antimicrobial resistance genes in environmental samples	3, 4
*2*. *Indicate* the advantage of using long-read sequence data over other methods when trying to match an antimicrobial resistance gene in a sample to the species from which it originated	4, 5
*3*. *Assess* the read length distribution of a metagenomic long-read sequencing run.	6
*4*. *Interpret* bioinformatics output to identify the dominant species present in a metagenomic DNA sample	7, 8, 9
*5*. *List* the steps required in bioinformatics to identify a resistance gene from an environmental sample and match it to its host species.	5
*6*. *Explain* how the concepts of evolutionary conservation and homology underly bioinformatics research	1, 2, 8
*7*. *Interpret* bioinformatics output to identify resistance genes and match them to their host species	8

^1^See Supporting Information (S3 File) for pre- and post- assessment questions.

By integrating core competencies of bioinformatics, students were exposed to emerging molecular methods in biology and environmental health [45]. The modular design of PARE-Seq was intended to make it useful in a wide range of courses, based on the instructor’s own curriculum.

### Assessment design

We opted to design our assessment to gauge understanding of the short bioinformatics and follow-up bioinformatics analysis activities only (Parts 2 and 3). We wanted to focus specifically on the bioinformatics activity since we are not aware of any other curricula using the Galaxy platform and because we felt this portion of the activity could be most challenging for students or instructors who were not prepared to teach bioinformatics due to the rapid transition to online learning precipitated by COVID. We understood that in so doing, we would likely decrease our potential to show gains, but this was a conscious decision for the above reasons. In other words, we did not want to inflate learning gains, potentially leading to the assumption that students were learning bioinformatics when instead, their learning was due to improved understanding of general antimicrobial resistance.

In developing the pre- and post-assessment, we used a multiple choice (MC) and/or true-false (T/F) format. Each question was designed to address specific learning objectives as well as a particular level of Bloom’s taxonomy, ranging from more basic understanding higher level of analytic thinking [44]. Questions spanned a range of material including application of knowledge from the bioinformatics activity to interpreting a figure or table analogous to output expected in the bioinformatics activity. In both the first (Fall 2020) and second (Spring 2021) iteration of PARE-Seq, it was suggested that students watch Part 1 videos, designed to provide them a baseline understanding of the concepts of AMR, One Health, and environmental surveillance, as students came from a range of institutions with differing knowledge on these topics. Next, students completed the pre-assessment after Part 1 videos (Fig 2). After completion of Parts 2 and 3, students were directed to take the post-assessment, consisting of the same questions, to ascertain their conceptual understanding and application of the content.

### Item analyses

Comparison of discrimination indices, point-biserial correlation, and Cronbach’s alpha values from the Fall 2020 and Spring 2021 cohorts indicated improvements to our assessment tool via the revisions made after the pilot (Tables 3 and 4). Desired value of the discrimination index for a question is > 0.3, and the average increased from 0.32 to 0.37, indicating the Spring 2021 assessment tool had greater ability to distinguish between high- and low-performing students [46, 47]. Point biserial correlations higher than 0.2 are desired, and their mean increased from 0.42 to 0.60 (all questions’ values > 0.2), indicating greater single-item reliability across the second iteration of the assessment. A higher Cronbach’s alpha, which measures consistency of a series of binomial data, demonstrates greater internal reliability of the assessment as a whole in its second iteration [48]. This measure increased from 0.238 in the pilot (Table 3) to 0.595 during Spring 2021 administration (Table 4).

**Table 3:** Fall 2020 pilot item analyses: Difficulty, reliability, and discriminatory power of the student post-survey.

Item	Sample size (n)	Index of difficulty^b^	Discrimination index^c^	Point-biserial correlation^d^
1	68	0.29	0.12	0.25
2	68	0.57	0.44	0.47
3	68	0.49	0.5	0.57
4	68	0.37	0.38	0.42
5	68	0.35	0.18	0.18
6	68	0.5	0.35	0.51
7	68	0.46	0.32	0.57
Desired values		0.3–0.9	≥0.3	≥0.2
Cronbach’s alpha^a^: 0.238				

^a^Cronbach’s alpha is a measure of internal reliability of the post-survey as a whole.

^b^The proportion of students who answered the question correctly.

^c^Indicates a question’s ability to distinguish between high-performing students and low performing students.

^d^Compares student performance on individual questions with their total scores, giving a measure of single-item reliability.

**Table 4:** Spring 2021 administration item analyses: Difficulty, reliability, and discriminatory power of the student post-assessment.

Item	Sample size (n)	Index of difficulty^b^	Discrimination index^c^	Point-biserial correlation^d^
1	101	0.5	0.308	0.385
2	101	0.625	0.288	0.478
3	101	0.529	0.52	0.697
4	101	0.635	0.385	0.680
5	101	0.760	0.404	0.841
6	101	0.548	0.404	0.65
7	101	0.452	0.327	0.531
8	101	0.625	0.365	0.558
9	101	0.346	0.346	0.606
10	101	0.798	0.327	0.587
Desired values		0.3–0.9	≥0.3	≥0.2
Cronbach’s alpha^a^	0.595			

^a^Cronbach’s alpha is a measure of internal reliability of the post-survey as a whole.

^b^The proportion of students who answered the question correctly.

^c^Indicates a question’s ability to distinguish between high-performing students and low performing students.

^d^Compares student performance on individual questions with their total scores, giving a measure of single-item reliability.

### Students show significant gains in learning after participation in PARE-Seq

**Fig 4:**
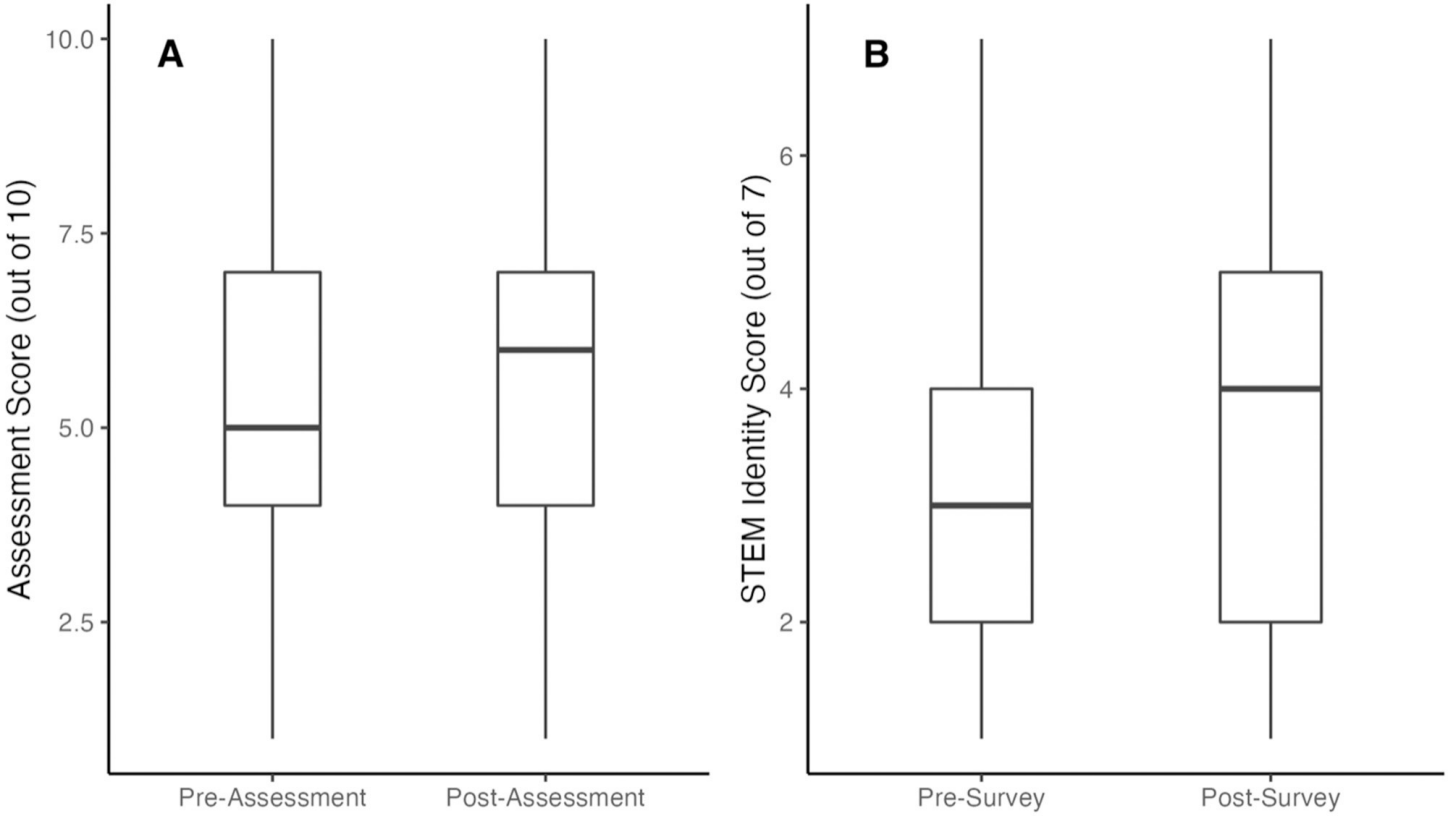
Student raw assessment score (A) and STEM identity score (B) distributions pre- and post- the bioinformatics activity of the PARE-Seq module (Spring 2021). Central bars represent median overall scores, boxes represent inner quartiles, and whiskers represent first and fourth quartiles. T-tests revealed significant differences between time points for both outcomes, indicating that students showed gains in learning and self-rated STEM identity after completing the bioinformatics modules of PARE-Seq.

We used the revised assessment instrument in the Spring 2021 cohort to measure learning gains. Across institutions, students had a mean learning score of 5.31 out of 10 (SD = 1.87) on the pre-assessment and 5.76 (SD = 2.23) out of 10 on the post-assessment (Fig 4, Table 5). This was a significant learning gain from pre- to post- intervention (t = 2.44, df = 100, *p* = .008). Despite reaching statistical significance, the effect size was relatively small (Cohen’s d = 0.22, 95% CI: -0.06, 0.50).

**Table 5:** Change in student assessment score pre- to post-module (learning gains) by student demographics.

Characteristic	Number of students (n)	Mean pre- score^a^	Mean post-score^a^	Mean change in assessment score
**Overall**	101	5.31	5.76	+0.51
*Institution (by Carnegie basic classification)*				
Doctoral/Professional University (Schools A and B)	84	5.48	6.09	+0.61
Associate’s College (School C)	7	4.43	3.71	-0.72
Baccalaureate/Associate’s College (School D)	10	5.50	6.20	+0.70
*Race/ethnicity*				
non-URM^b^	32	5.38	6.07	+0.69
URM	68	5.15	5.15	0
*Gender*				
Male	21	6.19	6.29	+0.1
Female	80	5.08	5.63	+0.55
*Extracurricular work hours*				
No extracurricular work	42	5.33	6.07	+0.74
0–10 hours	14	4.93	5.64	+0.71
11–20 hours	22	5.46	5.82	+0.36
21–30 hours	10	5.50	5.8	+0.30
31+ hours	12	5.17	4.83	-0.34
*Parental education*				
*4-year degree or more*	59	5.37	5.93	+0.56
*No parent graduated college*	*39*	5.26	5.49	+0.23
Self-rated quality of workspace				
*Few distractions/good quality (1–3)*	*69*	5.34	6.03	+0.69
*Many distractions/poor quality (4–6)*	*32*	5.22	5.19	-0.03

^a^Score out of 10 question assessment

^b^Underrepresented minority (Black/African American, Hispanic/Latine, Middle Eastern/North African, Biracial/Multiracial)

^c^1-6 rating scale

By institution type (Carnegie basic classification), students at Baccalaureate/Associate’s colleges had a mean score increase of 0.70 points, Doctoral/Professional university students had a mean increase of 0.61 points, but students at Associate’s only colleges (N = 7) had a mean decrease in score of 0.72 points. It is important to note here the small sample size of students from Associate’s only colleges and make any further conclusions by institution type with caution.

### Students showed significant gains in STEM identity after participation in PARE-Seq, but with small effect size

Student STEM identity score increased from pre- to post- intervention as well, with a mean of 3.19 (SD = 1.59) to 3.68 (SD = 1.71) out of 7 (Table 6). This increase was also statistically significant, indicating an overall increase in student STEM identity following completion of PARE-Seq (t = 3.92, df = 100, p < 0.001). Similar to learning gains, the effect size was relatively small (Cohen’s d = 0.30, 95% CI: 0.02, 0.57). We saw similar trends across institution types, as with learning gains: a mean increase of 0.3 points at Baccalaureate/Associate’s colleges, an increase of 0.59 points at Doctoral/Professional universities, but a decrease of 0.28 points at Associate’s only colleges (Table 6). Again, the number of students from Associate’s only colleges is very low.

**Table 6:** STEM identity score changes by student demographics.

Characteristic	Number of students (n)	Mean pre- rating^a^	Mean post-rating^a^	Mean change in STEM identity score
**Overall**	101	3.19	3.68	+0.49
*Institution (Carnegie basic classification)*				
Doctoral/Professional University (Schools A and B)	84	3.17	3.76	+0.59
Associate’s College (School C)	7	3.71	3.43	-0.28
Baccalaureate/Associate’s College (School D)	10	3.50	3.80	+0.30
*Race/ethnicity*				
non-URM^b^	32	3.41	3.86	+0.45
URM	68	2.75	3.34	+0.59
*Gender*				
Male	21	3.43	3.95	+0.52
Female	80	3.13	3.61	+0.48
*Extracurricular work hours*				
No extracurricular work	42	3.05	3.76	+0.71
0–10 hours	14	3.21	3.57	+0.36
11–20 hours	22	3.36	4.05	+0.69
21–30 hours	10	2.70	3.1	+0.31
31+ hours	12	3.83	3.50	- 0.33
*Parental education*				
*4-year degree or more*	59	3.31	3.81	+0.50
*No parent graduated college*	*39*	3.13	3.51	+0.38
Self-rated quality of workspace^c^				
*Few distractions/good quality (rating 4–6)*	*69*	3.30	3.87	+0.57
*Many distractions/poor quality (rating 1–3)*	*32*	2.94	3.28	+0.34

^a^Based on a 7-level rating scale

^b-c^See Table 5 for predictor descriptions.

Though not statistically significant and limited by small sample sizes, further investigation of demographic trends in STEM identity scores highlighted notable relationships (Table 6). Students self-identifying as underrepresented minority (Black/African American, Middle Eastern/North African, Hispanic/Latine, Native Hawaiian/Pacific Islander, and Biracial/Multiracial identifying students) had a mean score increase 0.14 points higher than non-URM (White and Asian students). Also of note, there was a decrease in STEM identity score from pre- to post-intervention—from 3.83 to 3.50 points—among those students working over 30 hours per week outside of academics. Male- and female-identifying students had similar rating increases (0.52 and 0.48 respectively, but males on average had higher post-module ratings (3.95 compared to 3.61).

### Student host institution is the strongest predictor of learning gains

While overall scores can detect broader patterns in student performance, we employed regression models to investigate changes in both outcomes (learning gains and STEM identity) as influenced by sociodemographic predictors. We first examined bivariate associations between coviariates and both outcomes of interest, and then report linear regression models for learning gains (Table 7) to estimate the contributions that various factors make to overall score. We found few statistically significant effects of covariates on either outcome. With respect to learning gains, student host institutions had a significant effect on score; those who attended School B, one of the two doctoral/professional universities, performed on average 1.08 points higher on their post-tests compared to the reference university, School A (*p* = 0.013).

**Table 7:** Bivariate associations and multiple linear regression model of the effect of student sociodemographic characteristics on learning gains.

	Bivariate Analysis			Multiple linear regression		
Predictor^a^	Estimate	SE	*p*	Estimate	SE	*p*
(Intercept)	-	-	-	0.28	0.68	0.683
Gender (ref: male)						
*Female*	0.45	0.46	0.326	0.77	0.5	0.128
Race/ethnicity (ref: non-URM^b^)						
*URM*	-0.69	0.40	0.087	-0.52	0.43	0.227
Parental education (ref: parent graduated college)						
*No parent graduated college*	-0.33	0.39	0.399	-0.01	0.43	0.976
Extracurricular work hours^c^ (ref: 0 hours/week)						
*1–10 hours*	-0.02	0.58	0.967	-0.36	0.63	0.567
*11–20 hours*	-0.37	0.49	0.450	-0.66	0.52	0.208
*21–30 hours*	-0.44	0.66	0.509	-0.78	0.71	0.275
*31+ hours*	-1.05	0.41	0.012*	-0.87	0.68	0.205
Self-rated quality of workspace^d^ (ref: few distractions/good quality)						
*Many distractions/poor quality*	-0.71	0.40	0.076	-0.42	0.48	0.384
Level of completion of PARE-Seq^e^ (ref: all components)						
*Some or all video lectures*	-0.24	0.50	0.630	-0.25	0.53	0.639
*All video lectures + bioinformatics activity*	-0.11	0.84	0.894	-0.12	1.02	0.904
*All components other than hypothesis-building discussion*	-0.54	0.67	0.421	-0.44	0.69	0.530
Student’s host institution (ref: School A^f^)						
*B*	1.01	0.40	0.014*	1.08	0.43	0.013*
*C*	0.60	0.63	0.346	1.36	0.84	0.109
*D*	-0.82	0.74	0.270	-0.47	0.88	0.594
Observations					101	
R^2^ / Adjusted R^2^					0.178 / 0.036	

*p<0.05

^a^Estimates for nominal variables indicate the modeled effect based on membership to the italicized group in comparison with the reference (ref) group.

^b^URM = underrepresented minority (see Table 6 for details).

^c^Work hours refers to students’ extracurricular work hours per week

^d^Rating question, 1–3 = poor quality, or many distractions, 4–6 = good quality, or very few distractions

^e^As PARE-Seq was constructed in a modular format, students may have not completed all components. All components include: all lectures, the Galaxy bioinformatics activity, a discussion on hypothesis building led by their instructor, and the final project.

^f^Student school abbreviated for anonymity. Institution with the largest number of students selected as reference group. By Carnegie basic classification, Schools A and B are doctoral/professional universities, C is an associate’s only college, and D is a baccalaureate/associate’s college.

In the bivariate model for learning gains, those working over 31 hours per week reported mean learning gains 1.05 points lower than others (*p* = 0.012), though this finding was not statistically significant when other covariates were introduced. Student gender also approached significance with respect to learning gains; students identifying as female had mean learning gains 0.77 points higher than males (*p =* 0.128). Race/ethnicity, parental education, self-rated quality of workspace, and self-reported level of completion of PARE-Seq did not appear to be significant predictors of learning gains.

### Student extracurricular work hours are the strongest predictor of STEM identity gains

As for learning gains, we employed regression models to investigate how changes in STEM identity were influenced by sociodemographic predictors (Table 8). For STEM identity scores, students working an extracurricular job over 31 hours per week reported significantly poorer outcomes; mean increase in STEM identity score was 0.95 points lower, controlling for covariates (*p* = 0.046).

**Table 8:** Bivariate assotiations and multiple linear regression model of the effect of student sociodemographic characteristics on STEM Identity score change.

	Bivariate Analysis			Multiple linear regression		
Predictor^a^	Estimate	SE	*p*	Estimate	SE	*p*
(Intercept)	-	-	-	0.69	0.47	0.146
Gender (ref: male)						
*Female*	-0.04	0.31	0.908	0.02	0.35	0.948
Race/ethnicity (ref: non-URM^b^)						
*URM*	0.14	0.27	0.617	0.25	0.3	0.401
Parental education (ref: parent graduated college)						
*No parent graduated college*	-0.12	0.26	0.630	-0.14	0.3	0.645
Extracurricular work hours^c^ (ref: 0 hours/week)						
*1–10 hours*	-0.36	0.39	0.359	-0.28	0.44	0.522
*11–20 hours*	-0.03	0.33	0.922	-0.03	0.36	0.942
*21–30 hours*	-0.31	0.44	0.478	-0.18	0.49	0.720
*3* *1+ hours*	-1.05	0.41	**0.012** *	-0.95	0.47	0.046*
Self-rated quality of workspace^d^ (ref: few distractions/good quality)						
*Many distractions/poor quality*	-0.16	0.36	0.663	0.13	0.33	0.696
Level of completion of PARE-Seq^e^ (ref: all components)						
*Some or all video lectures*	0.10	0.33	0.773	0.09	0.36	0.809
*All video lectures + bioinformatics activity*	-0.38	0.56	0.497	-0.29	0.71	0.684
*All components other than hypothesis-building discussion*	-0.45	0.45	0.312	-0.47	0.48	0.333
Student’s host institution (ref: *A*^f^)						
*B*	-0.02	0.28	0.942	-0.13	0.3	0.65
*C*	-0.29	0.44	0.509	-0.06	0.58	0.924
*D*	-0.88	0.51	0.090	-0.34	0.61	0.576
Observations					101	
R^2^ / Adjusted R^2^					0.109 / -0.044	

*p < 0.05.

^a-f^See Table 7 for predictor descriptions.

For both outcomes, we investigated interaction of covariates as well, to determine if there were compounding effects of particular student demographics. All interaction terms were screened in both models at a *p* < 0.05, but none were statistically significant.

## Discussion

### Making course-based research accessible and inclusive

To fill the identified gap in accessing research experiences for students during COVID-19, we developed the PARE-Seq activity to expose undergraduates to emerging molecular methods research, with applications to surveillance of AMR in the environment, but in an entirely online format that is adaptable to a range of classroom settings. The pedagogical decisions made in designing the course attempted to support underrepresented identities in STEM through student-led instruction, centering female teachers with different racial identities, and providing open access curricula and bioinformatics training.

To our knowledge, PARE-Seq represents the first freely available instrument teaching molecular methods for antimicrobial resistance detection in a virtual, modular format. Several distinguishing features make this course amenable to a wide range of institutions and learners. First, PARE-Seq makes high thruput, large computing power analysis of metagenomic data accessible to students through use of Galaxy, an open-source bioinformatics platform that runs workflows on cloud capacity rather than requiring a high-performance computing cluster. In addition, instructors may choose which portion of the curriculum best suits their course need, and provision of multiple formats for the analytic project (a lab report or poster presentation with assignment documentation and rubrics on the host site) allows them to assess their students’ learning in the method most appropriate. Finally, the subject matter of PARE-Seq being bioinformatics has been well substantiated as a field particularly well-suited for virtual learning, as iterations can be performed rapidly and aren’t cost intensive [30].

Future developments to the PARE-Seq course have the potential to bring environmental surveillance to a more real and applicable format. With time and resources, this course can be adapted for students to collect their own soil and water samples and sequence them in the lab, followed by utilization of the same bioinformatics workflow introduced in the current module. Studies have already demonstrated the use of Oxford Nanopore sequencing technology in a classroom setting, a cutting-edge approach to teaching students emerging metagenomic methods [49]. Finally, PARE-Seq can be further incorporated into other existing PARE modules, such as through sequencing isolates from the existing library modules [16, 50]. These adaptations would engage students to an even more realistic degree in the environmental surveillance of AMR.

### Students value diverse teacher identities, flexible learning opportunities

Student feedback from post-course surveys echoed quantitative findings of this study, and gave us qualitative insight to the impact of the pedagogical decisions made while developing PARE-Seq. Student comments included the following:

*“I love how it was all women teaching.”*

*“Very easy to understand and helpful! Seeing women and POC in STEM does make a difference even if it is not talked about.”*

*“Great job! As you are students, you made the information much easier to understand and relate to.”*

*“They did a great job as they made antibiotic resistance to be comprehended in an easy way.”*

These comments suggest that participants found value in learning from fellow students from underrepresented identities in STEM fields, a potential explanation for the overall gains we observed in STEM identity. The impact of peer-learning, female-identifying instructors, and those from marginalized race/ethnicities is well documented but their synergistic effect is less understood [40, 41, 51].This study indicates the value of such pedagogical decisions to increase equity in STEM education, and future research should build on this finding.

### Intervention outcomes

PARE-Seq students experienced both significant learning gains and increase in STEM identity scores over the course of this short program. However, effect sizes were small, perhaps owing to the very short term nature of the intervention.

#### Extracurricular work hours

A student’s extracurricular work hours were a predictor of both learning gains and STEM identity. Though only marginally significant, students working at a job outside of academics over 30 hours per week had an average change in learning assessment score 0.87 points lower than students who reported no extracurricular work. Students working over 30 hours per week had 0.95 point lower changes in STEM identity (out of a score of 7) than those who did not. Examination of the trends in mean scores pre-to post-course by covariates (Table 6) indicates that students working over 30 hours per week were the only group who reported a reduction in their confidence as a STEM professional from pre- to post-assessment. It is interesting that both URM and >10 work hours/week are negative predictors for learning gains but only the 31+ hour group also shows a decrease in STEM identity score. This could point to the benefit of racially diverse teaching. Perception of competence, or self-efficacy in STEM skills, is thought to play a role in development of STEM identity [22, 52]. Low or no learning gains may be driving a lower STEM identity score in both groups, but a diverse teaching team may neutralize this effect in URM students. Repeating this intervention with a control teaching team may help to answer this question, but we acknowledge the relationship between self-efficacy, performance and STEM identity is complex.

Growth in student loan debt, cost of higher education, and federal borrowing for education over the past two decades has grown significantly, and many undergraduates are working while enrolled in school [53, 54]. This finding brings to light the challenge of needing to work while in college and its potential impact on a student’s ability to learn. In addition, our finding reinforces the importance of providing a stipend for apprentice-style (out-of-class) undergraduate research opportunities for STEM students [55]. Minimal literature to date investigates the effect of extracurricular employment on STEM identity or course performance [56]. We suggest that support mechanisms for students who must seek of extracurricular employment during college should be further explored and prioritized to promote equity in the classroom.

#### Demographic factors

Though only marginally significant, female-identifying students had larger learning gains than male-identifying students by an average of 0.44 points, out of a score of 10. Substantive literature demonstrates the value of female mentors in contributing to female student success in STEM, and PARE-Seq’s design resulting in a similar outcome supports this conclusion [42, 43]. However, this sample was imbalanced by gender, so these results should be interpreted with caution. Students identifying as Black or African American, Middle Eastern or North African, Hispanic/Latine, Native Hawiian/Pacific Islander or Multiracial (combined), on average, had increases in STEM identity scores 0.14 points higher than White or Asian-identifying students, but had mean score increases on the post-(learning) assessment of 0.58 points lower than non-underrepresented minorities in STEM (White and Asian students). Without a control, we cannot know how these scores would differ if we had not included undergraduate female-identifying or a woman of color as instructors in the video series. Though we cannot assign a causal relationship, this finding may be indicative that our pedagogical prioritization of highlighting teachers and expert interviews with different racial identities had positive influence on STEM identity among URM students. This trend is substantiated by prior work on factors that improve STEM confidence [1, 42, 51, 57].

#### Institution

Influence of institution type on learning gains (change in assessment score pre- to post-course) may be attributable to fidelity of implementation such as differences in teaching style or curriculum adaptation. Alternatively, student preparedness, and the level or type of course in which students at each institution were completing PARE-Seq may play a role. The overall decrease in learning gains observed in students at Associate’s institutions raises concerns about the efficacy of this bioinformatics activity with this target audience; however the sample size in this group was very small. Student host institution was not a significant predictor of STEM identity. This finding indicates PARE-Seq’s potential to impact student STEM confidence independent of their institution, which adds to the potential scalability of the course.

### Limitations

Given the desire for a rapid dissemination of the module in the context of the COVID-19 pandemic, our assessment tool is somewhat limited by a lower-than-desired Cronbach’s alpha, despite significant work put into designing and iterating the tool during the pilot phase of the project. A Cronbach’s alpha of >0.6 is generally desired, so our value of 0.595 is a limitation of this study. Any future user of this instrument may wish to revise items 1 and 5, each of which have low point-biserial correlation and discrimination index values. In addition, since Cronbach’s alpha generally increases as the number of items increases, adding more items to the assessment may improve reliability score.

It is important to note STEM identity outcomes were assessed through only the previously validated single-item measure of STEM identity [24]. Though rigorously developed, it is one question, asked over a short duration of intervention, and therefore may lose some of the dimensions to this complex concept for students who are in the process of developing their career interests and identity as scientists. During survey development we considered multiple methods to assess this outcome, including open-response and multi-part Likert scale questions, but for both analysis potential and the threat of student survey fatigue, we decided to employ this single-question approach.

Though students at 12 institutions participated in either the pilot or administration phases of PARE-Seq, many instructors chose not to require student participation in the assessment, so our analysis misses comprehensive data on the student population and may be affected by non-responses. The decision to combine race/ethnicity identities into a binary variable for analyses was made to avoid issues with small sample numbers and low power of the study, but we recognize that no sociodemographic identities should be treated as a binary, and future studies can recruit a larger sample size to avoid this. Finally, a larger and more balanced sample by institution type and gender could have allowed us to better identify learning outcome trends, particularly since PARE-Seq aims to provide research experiences for students at non-Doctoral granting institutions, where they may be limited. In future iterations, we recommend developing a mechanism to make assessments required.

## Conclusion

This study was motivated by new methodologies in sequencing-based environmental surveillance of AMR and corresponding bioinformatic analyses, a lack of research opportunities for undergraduates during the COVID-19 pandemic, and the need to develop teaching resources that empower underrepresented identities in STEM fields. In response, we developed PARE-Seq as an open-source CURE at the intersection of bioinformatics and public health pedagogy. Our study indicates that students exhibited both learning gains and increases in STEM confidence through participation in this short intervention. Learning gains were significantly associated with student host institution. Though not statistically significant, other potential relationships were revealed; female-identifying students saw greater learning gains than males, and students identifying as an underrepresented minority reported larger increases in STEM identity score. This may suggest a positive impact on students with these identities when learning from a diverse, female-led teaching team and warrants further exploration. Increases in STEM identity were hindered for students with high extracurricular work hours, highlighting that there was a need to support students working jobs outside of the classroom during the COVID-19 pandemic. These findings demonstrate that even short interventions have the potential to yield learning gains and improve student confidence in pursuing STEM, but support must be prioritized for students working outside of school. By providing ready-to use curricula like PARE-Seq, we can better equip STEM instructors to utilize research-driven learning resources that improve outcomes for all students.

### How to obtain and administer PARE-Seq

PARE-Seq is housed at an online portal (www.pareseq.com) or through the PARE website (https://sites.tufts.edu/ctse/pare/) where interested students or instructors can access and coordinate administration of PARE-Seq. The host site provides a course overview video, directs students to video lectures and necessary materials, and supplies instructors with information for teaching the course. The pre- and post- assessment is available in Supplementary Materials. Users can make a Galaxy account following the information provided on the online portal. Users wishing to conduct research using PARE-Seq should contact the corresponding author for more information on data accessibility.

## Supporting information

S1 File PARE-Seq instructor post-survey. Administered to teachers who participated in the Fall 2020 pilot of the module. Findings were used for iteration of course material before Spring 2021 administration.
(DOCX)

S2 File PARE-Seq student feedback survey. Administered to students who participated in the Fall 2020 pilot of the PARE-Seq module. Findings were used for iteration of course material before Spring 2021 administration.
(DOCX)

S3 File PARE-Seq pre/post-assessment and post-survey. Includes consent form, sociodemographic questions (Sections 1 and 3) and assessment (Section 2). The same assessment was administered to students pre- and post-completion of the bioinformatics modules.
(DOCX)

## References

[1] Carpi A, Ronan DM, Falconer HM, Lents NH. Cultivating minority scientists: Undergraduate research increases self-efficacy and career ambitions for underrepresented students in STEM. J Res Sci Teach. 2017;54(2):169–94.

[2] Krim JS, Coté LE, Schwartz RS, Stone EM, Cleeves JJ, Barry KJ, et al. Models and Impacts of Science Research Experiences: A Review of the Literature of CUREs, UREs, and TREs. CBE—Life Sci Educ. 2019 Dec;18(4):ar65. doi: 10.1187/cbe.19-03-0069 31782694 PMC6889846

[3] Russell SH, Hancock MP, McCullough J. Benefits of Undergraduate Research Experiences. Science. 2007 Apr 27;316(5824):548–9. 17463273 10.1126/science.1140384

[4] Zydney AL, Bennett JS, Shahid A, Bauer KW. Impact of Undergraduate Research Experience in Engineering. J Eng Educ. 2002;91(2):151–7.

[5] Bangera G, Brownell SE. Course-Based Undergraduate Research Experiences Can Make Scientific Research More Inclusive. CBE—Life Sci Educ. 2014 Dec;13(4):602–6. doi: 10.1187/cbe.14-06-0099 25452483 PMC4255347

[6] Elgin SCR, Bangera G, Decatur SM, Dolan EL, Guertin L, Newstetter WC, et al. Insights from a Convocation: Integrating Discovery-Based Research into the Undergraduate Curriculum. CBE—Life Sci Educ. 2016 Jun;15(2):fe2. doi: 10.1187/cbe.16-03-0118 27146158 PMC4909350

[7] Estrada M, Burnett M, Campbell AG, Campbell PB, Denetclaw WF, Gutiérrez CG, et al. Improving Underrepresented Minority Student Persistence in STEM. CBE—Life Sci Educ. 2016 Sep;15(3):es5. doi: 10.1187/cbe.16-01-0038 27543633 PMC5008901

[8] Wei CA, Woodin T. Undergraduate Research Experiences in Biology: Alternatives to the Apprenticeship Model. CBE—Life Sci Educ. 2011 Jun;10(2):123–31. doi: 10.1187/cbe.11-03-0028 21633057 PMC3105915

[9] Esparza D, Wagler AE, Olimpo JT. Characterization of Instructor and Student Behaviors in CURE and Non-CURE Learning Environments: Impacts on Student Motivation, Science Identity Development, and Perceptions of the Laboratory Experience. CBE—Life Sci Educ. 2020 Mar;19(1):ar10. doi: 10.1187/cbe.19-04-0082 32108560 PMC8697643

[10] Frantz KJ, Demetrikopoulos MK, Britner SL, Carruth LL, Williams BA, Pecore JL, et al. A Comparison of Internal Dispositions and Career Trajectories after Collaborative versus Apprenticed Research Experiences for Undergraduates. CBE—Life Sci Educ. 2017 Mar;16(1):ar1. doi: 10.1187/cbe.16-06-0206 28130268 PMC5332035

[11] Shuster M, Curtiss J, Wright T, Champion C, Sharifi M, Bosland J. Implementing and Evaluating a Course-Based Undergraduate Research Experience (CURE) at a Hispanic-Serving Institution. Interdiscip J Probl-Based Learn [Internet]. 2019 Aug 30;13(2). Available from: https://docs.lib.purdue.edu/ijpbl/vol13/iss2/1

[12] Corwin LA, Graham MJ, Dolan EL. Modeling Course-Based Undergraduate Research Experiences: An Agenda for Future Research and Evaluation. CBE—Life Sci Educ. 2015 Mar 2;14(1):es1. doi: 10.1187/cbe.14-10-0167 25687826 PMC4353087

[13] Corwin LA, Runyon CR, Ghanem E, Sandy M, Clark G, Palmer GC, et al. Effects of Discovery, Iteration, and Collaboration in Laboratory Courses on Undergraduates’ Research Career Intentions Fully Mediated by Student Ownership. CBE—Life Sci Educ. 2018 Jun;17(2):ar20. doi: 10.1187/cbe.17-07-0141 29749845 PMC5998318

[14] Dolan E. Course-based undergraduate research experiences: Current knowledge and future directions. Wash DC Natl Res Counc [Internet]. 2016; Available from: https://sites.nationalacademies.org/cs/groups/dbassesite/documents/webpage/dbasse_177288.pdf

[15] Linn MC, Palmer E, Baranger A, Gerard E, Stone E. Undergraduate research experiences: Impacts and opportunities. Science. 2015 Feb 6;347(6222):1261757. 25657254 10.1126/science.1261757

[16] Genné-Bacon EA, Bascom-Slack CA. The PARE Project: A Short Course-Based Research Project for National Surveillance of Antibiotic-Resistant Microbes in Environmental Samples. J Microbiol Biol Educ. 2018 Oct 31;19(3):19.3.97. doi: 10.1128/jmbe.v19i3.1603 30377474 PMC6203630

[17] Pokhrel S, Chhetri R. A Literature Review on Impact of COVID-19 Pandemic on Teaching and Learning. High Educ Future. 2021 Jan 1;8(1):133–41.

[18] Dooley DG, Simpson JN, Beers NS. Returning to School in the Era of COVID-19. JAMA Pediatr. 2020 Nov 1;174(11):1028–9. doi: 10.1001/jamapediatrics.2020.3874 32797162

[19] Burmeister AR, Dickinson K, Graham MJ. Bridging Trade-Offs between Traditional and Course-Based Undergraduate Research Experiences by Building Student Communication Skills, Identity, and Interest. J Microbiol Biol Educ [Internet]. 2021 Jun 30 [cited 2022 Mar 1]; Available from: https://journals.asm.org/doi/abs/10.1128/jmbe.00156-21. 10.1128/jmbe.00156-21 PMC8442013 34594446

[20] Medicine NA of S Engineering, and, Education D of B and SS and, Studies D on E and L, Curriculum C for C on IDBR into the U. Integrating Discovery-Based Research into the Undergraduate Curriculum: Report of a Convocation. National Academies Press; 2015. 161 p.

[21] Fry R, Kennedy B, Funk C. STEM Jobs See Uneven Progress in Increasing Gender, Racial and Ethnic Diversity [Internet]. Pew Research Center Science & Society. 2021 [cited 2022 Feb 21]. Available from: https://www.pewresearch.org/science/2021/04/01/stem-jobs-see-uneven-progress-in-increasing-gender-racial-and-ethnic-diversity/.

[22] Carlone HB, Johnson A. Understanding the science experiences of successful women of color: Science identity as an analytic lens. J Res Sci Teach. 2007;44(8):1187–218.

[23] Sfard A, Prusak A. Telling Identities: In Search of an Analytic Tool for Investigating Learning as a Culturally Shaped Activity. Educ Res. 2005 May 1;34(4):14–22.

[24] McDonald MM, Zeigler-Hill V, Vrabel JK, Escobar M. A Single-Item Measure for Assessing STEM Identity. Front Educ. 2019 Jul 26;4:78.

[25] Magana AJ, Taleyarkhan M, Alvarado DR, Kane M, Springer J, Clase K. A Survey of Scholarly Literature Describing the Field of Bioinformatics Education and Bioinformatics Educational Research. CBE—Life Sci Educ. 2014 Dec;13(4):607–23. doi: 10.1187/cbe.13-10-0193 25452484 PMC4255348

[26] Buonaccorsi VP, Boyle MD, Grove D, Praul C, Sakk E, Stuart A, et al. GCAT-SEEKquence: Genome Consortium for Active Teaching of Undergraduates through Increased Faculty Access to Next-Generation Sequencing Data. CBE—Life Sci Educ. 2011 Dec;10(4):342–5. doi: 10.1187/cbe.11-08-0065 22135368 PMC3228652

[27] Kovarik DN, Patterson DG, Cohen C, Sanders EA, Peterson KA, Porter SG, et al. Bioinformatics education in high school: implications for promoting science, technology, engineering, and mathematics careers. CBE Life Sci Educ. 2013;12(3):441–59. doi: 10.1187/cbe.12-11-0193 24006393 PMC3763012

[28] Machluf Y, Gelbart H, Ben-Dor S, Yarden A. Making authentic science accessible—the benefits and challenges of integrating bioinformatics into a high-school science curriculum. Brief Bioinform. 2017 Jan 1;18(1):145–59. doi: 10.1093/bib/bbv113 26801769 PMC5221422

[29] Madlung A. Assessing an effective undergraduate module teaching applied bioinformatics to biology students. PLOS Comput Biol. 2018 Jan 11;14(1):e1005872. doi: 10.1371/journal.pcbi.1005872 29324777 PMC5764237

[30] Lopatto D, Rosenwald AG, DiAngelo JR, Hark AT, Skerritt M, Wawersik M, et al. Facilitating Growth through Frustration: Using Genomics Research in a Course-Based Undergraduate Research Experience. J Microbiol Biol Educ [Internet]. 2020 [cited 2022 Feb 16]; Available from: https://journals.asm.org/doi/abs/10.1128/jmbe.v21i1.2005. 10.1128/jmbe.v21i1.2005 PMC7048401 32148609

[31] Anjum MF, Zankari E, Hasman H. Molecular Methods for Detection of Antimicrobial Resistance. Microbiol Spectr. 2017 Dec 7;5(6):5.6.02. doi: 10.1128/microbiolspec.ARBA-0011-2017 29219107 PMC11687549

[32] Franklin AM, Brinkman NE, Jahne MA, Keely SP. Twenty-first century molecular methods for analyzing antimicrobial resistance in surface waters to support One Health assessments. J Microbiol Methods. 2021 May 1;184:106174. doi: 10.1016/j.mimet.2021.106174 33774111 PMC8159016

[33] Murray CJ, Ikuta KS, Sharara F, Swetschinski L, Aguilar GR, Gray A, et al. Global burden of bacterial antimicrobial resistance in 2019: a systematic analysis. The Lancet. 2022 Feb 12;399(10325):629–55. 10.1016/S0140-6736(21)02724-0 PMC8841637 35065702

[34] Alvarez-Uria G, Gandra S, Mandal S, Laxminarayan R. Global forecast of antimicrobial resistance in invasive isolates of Escherichia coli and Klebsiella pneumoniae. Int J Infect Dis. 2018 Mar;68:50–3. doi: 10.1016/j.ijid.2018.01.011 29410253 PMC5889426

[35] O’Neill J. Tackling drug-resistant infections globally: final report and recommendations [Internet]. Government of the United Kingdom; 2016 May [cited 2022 Feb 18]. Available from: https://apo.org.au/node/63983.

[36] Allen MJ, Yen WM. Introduction to Measurement Theory. Waveland Press; 2001. 321 p.

[37] Ouimet JA, Bunnage JC, Carini RM, Kuh GD, Kennedy J. Using Focus Groups, Expert Advice, and Cognitive Interviews to Establish the Validity of a College Student Survey. Res High Educ. 2004 May 1;45(3):233–50.

[38] Wickham H. ggplot2: Elegant Graphics for Data Analysis. [Internet]. Springer-Verlag New York.; 2016. Available from: https://github.com/tidyverse/ggplot2.

[39] Torchiano M. effsize: Efficient Effect Size Computation [Internet]. 2020 [cited 2022 Dec 29]. Available from: https://CRAN.R-project.org/package=effsize.

[40] Buck G, Cook K, Quigley C, Eastwood J, Lucas Y. Profiles of Urban, Low SES, African American Girls’ Attitudes Toward Science: A Sequential Explanatory Mixed Methods Study. J Mix Methods Res. 2009 Oct 1;3(4):386–410.

[41] Jr D, Sawyer D III. Informing Higher Education Policy and Practice Through Intersectionality. J Progress Policy Pract. 2014 Jan 1;2.

[42] Moghe S, Baumgart K, Shaffer JJ, Carlson KA. Female mentors positively contribute to undergraduate STEM research experiences. PLOS ONE. 2021 Dec 2;16(12):e0260646. 10.1371/journal.pone.0260646 PMC8638905 34855824

[43] Raabe IJ, Boda Z, Stadtfeld C. The Social Pipeline: How Friend Influence and Peer Exposure Widen the STEM Gender Gap. Sociol Educ. 2019 Apr 1;92(2):105–23.

[44] Anderson LW, Krathwohl DR. A taxonomy for learning, teaching, and assessing: a revision of Bloom’s taxonomy of educational objectives. Complete ed. New York: Longman; 2001.

[45] Welch L, Lewitter F, Schwartz R, Brooksbank C, Radivojac P, Gaeta B, et al. Bioinformatics Curriculum Guidelines: Toward a Definition of Core Competencies. PLOS Comput Biol. 2014 Mar 6;10(3):e1003496. doi: 10.1371/journal.pcbi.1003496 24603430 PMC3945096

[46] Doran RL. Basic Measurement and Evaluation of Science Instruction [Internet]. National Science Teachers Association, 1742 Connecticut Ave; 1980 [cited 2022 Feb 18]. Available from: https://eric.ed.gov/?id=ED196733.

[47] Findley WG. A Rationale for Evaluation of Item Discrimination Statistics. Educ Psychol Meas. 1956 Jul 1;16(2):175–80.

[48] Stefanski KM, Gardner GE, Seipelt-Thiemann RL. Development of a Lac Operon Concept Inventory (LOCI). CBE Life Sci Educ. 2016;15(2):ar24. doi: 10.1187/cbe.15-07-0162 27252300 PMC4909346

[49] Zaaijer S, Columbia University Ubiquitous Genomics 2015 class, Erlich Y. Using mobile sequencers in an academic classroom. Shailes S, editor. eLife. 2016 Apr 7;5:e14258. 27054412 10.7554/eLife.14258 PMC4869913

[50] Fuhrmeister ER, Larson JR, Kleinschmit AJ, Kirby JE, Pickering AJ, Bascom-Slack CA. Combating Antimicrobial Resistance Through Student-Driven Research and Environmental Surveillance. Front Microbiol [Internet]. 2021 [cited 2022 May 17];12. Available from: https://www.frontiersin.org/article/10.3389/fmicb.2021.577821. doi: 10.3389/fmicb.2021.577821 33679626 PMC7931799

[51] Ireland DT, Freeman KE, Winston-Proctor CE, DeLaine KD, McDonald Lowe S, Woodson KM. (Un)Hidden Figures: A Synthesis of Research Examining the Intersectional Experiences of Black Women and Girls in STEM Education. Rev Res Educ. 2018 Mar 1;42(1):226–54.

[52] Herrera F. A., Hurtado S., Garcia G. A., and Gasiewski J. A Model for Redefining STEM Identity For Talented STEM Graduate Students [Internet]. 2012. Available from: http://www.heri.ucla.edu/nih/downloads/ AERA2012HerreraGraduateSTEMIdentity.pdf.

[53] Board of Governors of the Federal Reserve System (US). Student Loans Owned and Securitized [Internet]. FRED, Federal Reserve Bank of St. Louis. FRED, Federal Reserve Bank of St. Louis; 2006 [cited 2022 Feb 19]. Available from: https://fred.stlouisfed.org/series/SLOAS.

[54] Digest of Education Statistics, 2018 [Internet]. National Center for Education Statistics; [cited 2022 Feb 19]. Available from: https://nces.ed.gov/programs/digest/d18/tables/dt18_503.40.asp.

[55] McHugh PP. The impact of compensation, supervision and work design on internship efficacy: implications for educators, employers and prospective interns. J Educ Work. 2017 May 19;30(4):367–82.

[56] Henley L, Roberts P. Perceived Barriers to Higher Education in STEM Among Disadvantaged Rural Students: A Case Study. Inq J Va Community Coll [Internet]. 2016 Jan 1;20(1). Available from: https://commons.vccs.edu/inquiry/vol20/iss1/4.

[57] Kim AY, Sinatra GM. Science identity development: an interactionist approach. Int J STEM Educ. 2018 Nov 30;5(1):51. doi: 10.1186/s40594-018-0149-9 30631740 PMC6310440

