# Supplementary material for: Learning and STEM identity gains from an online module on sequencing-based surveillance of antimicrobial resistance in the environment: An analysis of the PARE-Seq curriculum
Source: PLoS One. 2023 Mar 10;18(3):e0282412. doi: 10.1371/journal.pone.0282412 (PMC10004520; doi:10.1371/journal.pone.0282412)
Supplement: S1 File — Administered to teachers who participated in the Fall 2020 pilot of the module. Findings were used for iteration of course material before Spring 2021 administration. (DOCX) [file pone.0282412.s001.docx]

**S1**

PARE-Seq Instructor Post-Survey. Administered to teachers who participated in the Fall 2020 pilot of the module. Findings were used for iteration of course material before Spring 2021 administration.

**TUFTS UNIVERSITY** **CONSENT TO PARTICIPATE IN A RESEARCH STUDY**

Title of the Study:  Evaluation of PARE-Seq: A Short Course on the Bioinformatics of Antibiotic Resistance

Principal Investigator: Scarlet Bliss, Tufts University

Faculty Advisor Carol Bascom-Slack, PhD, Tufts University

Phone: 617-636-3412

Email: scarlet.bliss@tufts.edu carol.bascom_slack@tufts.edu

You are being asked to volunteer in a research study. Please find below information about this research for you to carefully consider when deciding about whether or not to participate. Please ask questions about any of the information you do not understand before you decide whether to participate.  

**Key Information for You to Consider**

**Statement of Research** You are being asked to volunteer for a research study.  It is up to you whether you choose to participate or not.  There will be no penalty or loss of benefits if you choose not to participate or discontinue participation.

**Purpose**. The purpose of this research is to determine how the PARE-Seq short course can be improved, to evaluate the effectiveness of our curriculum, and to address research questions relating to student experiences with online learning.

**Duration.** It is expected that your participation will take 10 minutes

**Procedures and Activities.** This survey is intended for completion after your instruction of PARE-Seq. The survey consists of multiple choice, Likert-style, and open response questions that ask about your teaching structure, experience and programmatic feedback for improvement of the course in future iterations. Participation is voluntary and you may choose to not respond to any questions or leave the survey at any time.

**Risks:** There is minimal risk associated with participation.

**Benefits:** There are no personal benefits for participation. You will not receive any compensation for your participation in this study.

Do you authorize Tufts University researchers to use your responses to improve the quality of the course for future iterations?

- Yes
- No

I taught the PARE-Seq short course

- In an in-person course
- Online in a remote course
- In a hybrid course (partially in-person, partially online)

Please select all materials/tools you utilized from the course:

- Video 1: Understanding Antibiotics and Antibiotic Resistance
- Video 2: Antimicrobial Resistant Bacteria Spread - through the environment and to our bodies
- Video 3: Why is Antimicrobial Resistance a Public Health Issue?
- Video 4: Spotlight on Antimicrobial Resistance Genes of Clinical Significance
- Video 5: Tools for Bioinformatics - how to we find a needle in a haystack?
- Video 6: Analyzing a Metagenomic Sample in Galaxy
- Galaxy Video Tutorial
- Galaxy Activity
- Final Lab Report
- Final Poster Presentation

What, if any, component of the PARE-Seq short course was confusing or challenging to teach?

- The video lectures
- The Galaxy activity
- The final lab report
- The final poster presentation
- None of these components were confusing or challenging to teach

Please comment on the appropriateness and quality of the *video lectures*. Did they support your teaching of PARE-Seq concepts?

________________________________________________________________

Please comment on the appropriateness and quality of the *Galaxy activity*. Did they support your teaching of PARE-Seq concepts?

________________________________________________________________

How do you perceive the impact of learning from a diverse teaching staff on your students' interest in the material and/or STEM?

- Negatively impacted their learning
- No impact on their learning
- Positively impacted their learning

How do you perceive the impact of learning from fellow college students on your students' interest in the material and/or STEM?

- Negatively impacted their learning
- No impact on their learning
- Positively impacted their learning

*Please indicate the access to technology you believe your students had when participating in online coursework. (Later, we will ask about their participation in PARE-Seq).*


The majority of my students:

|  | Agree | Not sure | Disagree |
| --- | --- | --- | --- |
| have sufficient access to an electronic device for the online coursework |  |  |  |
| use a shared computer or electronic device for their online coursework |  |  |  |
| have exclusive access to their own electronic device for their online coursework |  |  |  |
| use a phone or tablet for their online coursework |  |  |  |
| use a laptop or desktop computer, or a Chromebook for their online coursework. |  |  |  |

How many of your students do you believe had to seek technology different than what they normally use (as indicated above) for their participation in PARE-Seq?

- None
- A few
- Less than half of my students
- The majority of my students
- I don't know

*Consider the availability of Wi-Fi for your students while participating in PARE-Seq.*
I believe the majority of my students:

|  | Agree | Not sure | Disagree |
| --- | --- | --- | --- |
| were able to access sufficient Wi-Fi |  |  |  |
| had access to sufficient Wi-Fi in their residence |  |  |  |
| had to travel outside of their residence to access sufficient Wi-Fi |  |  |  |
| had difficulty accessing sufficient Wi-Fi |  |  |  |

Did PARE-Seq change your confidence in teaching bioinformatics concepts to your students?

- Yes
- No
- Unsure

Do you believe PARE-Seq improved your students' understanding of bioinformatics this term?

- Yes
- No
- Unsure

How does PARE-Seq compare to other methods you've used to teach bioinformatics?

- Better
- Worse
- I have not taught bioinformatics before

Overall, what would improve the PARE-Seq short course, or your teaching of the material to your students?

________________________________________________________________
