# Supplementary material for: Learning and STEM identity gains from an online module on sequencing-based surveillance of antimicrobial resistance in the environment: An analysis of the PARE-Seq curriculum
Source: PLoS One. 2023 Mar 10;18(3):e0282412. doi: 10.1371/journal.pone.0282412 (PMC10004520; doi:10.1371/journal.pone.0282412)
Supplement: S2 File — Administered to students who participated in the Fall 2020 pilot of the PARE-Seq module. Findings were used for iteration of course material before Spring 2021 administration. (DOCX) [file pone.0282412.s002.docx]

**S2**

**PARE-Seq Student Feedback Survey.** Administered to students who participated in the Fall 2020 pilot of the PARE-Seq module. Findings were used for iteration of course material before Spring 2021 administration.

In my class, I completed (select one)

- Some of the video lectures
- All of the video lectures
- All lectures and Galaxy activity
- All lectures, Galaxy activity, and final assignment (lab report)
- All lectures, Galaxy activity, and final assignment (poster presentation)
- Other (please specify) __________________________________________________

The amount of time I spent doing the PARE-Seq short course was

- One class period
- Two class periods in the same week
- Two class periods in separate weeks
- More than two class periods (please explain) __________________________________________________

Please indicate your level of agreement with the following statements 
(1 = strongly disagree, 6 = strongly agree)

|  | 1 | 2 | 3 | 4 | 5 | 6 |
| --- | --- | --- | --- | --- | --- | --- |
| The difficulty level of information was appropriate |  |  |  |  |  |  |
| The pace of information was appropriate |  |  |  |  |  |  |
| I understood the material in the video lectures |  |  |  |  |  |  |
| The video lectures were interesting to watch |  |  |  |  |  |  |
| I understood how to do the Galaxy activity after participating in PARE-Seq fully |  |  |  |  |  |  |
| In video lectures, the information was presented too rapidly |  |  |  |  |  |  |
| The Galaxy activity contributed to my learning |  |  |  |  |  |  |
| I enjoyed the Galaxy activity |  |  |  |  |  |  |

Did you notice diversity in the teaching staff for this short course?

- Yes
- No

How impactful is learning from a diverse teaching staff on your interest in the material/STEM? (1 = not at all impactful, 6 = extremely impactful)

|  | 1 | 2 | 3 | 4 | 5 | 6 |
| --- | --- | --- | --- | --- | --- | --- |
| Learning from a diverse teaching staff |  |  |  |  |  |  |

How valuable were these aspects of the PARE-Seq course?
(1 = not at all valuable/informative, 6 = very valuable/informative)

|  | 1 | 2 | 3 | 4 | 5 | 6 | N/A (Didn't complete) |
| --- | --- | --- | --- | --- | --- | --- | --- |
| Video lectures |  |  |  |  |  |  |  |
| Being taught by fellow university students |  |  |  |  |  |  |  |
| The Galaxy activity |  |  |  |  |  |  |  |
| Interviews with professionals in the field |  |  |  |  |  |  |  |
| The final presentation or lab report |  |  |  |  |  |  |  |

Do you have anything you'd like to tell us about your experience in PARE-Seq?

________________________________________________________________

Overall, I would rate my experience of the PARE-Seq online learning lab

| (1 star = lowest, 5 stars = highest rating) | 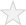 | 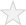 | 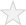 | 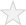 | 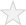 |
| --- | --- | --- | --- | --- | --- |
