# Supplementary material for: Learning and STEM identity gains from an online module on sequencing-based surveillance of antimicrobial resistance in the environment: An analysis of the PARE-Seq curriculum
Source: PLoS One. 2023 Mar 10;18(3):e0282412. doi: 10.1371/journal.pone.0282412 (PMC10004520; doi:10.1371/journal.pone.0282412)
Supplement: S3 File — Includes consent form, sociodemographic questions (Sections 1 and 3) and assessment (Section 2). The same assessment was administered to students pre- and post-completion of the bioinformatics modules. (DOCX) [file pone.0282412.s003.docx]

**S3**

**PARE-Seq Pre/Post-Assessment and Post-Survey.** Includes consent form, sociodemographic questions (Sections 1 and 3) and assessment (Section 2). The same assessment was administered to students pre- and post-completion of the bioinformatics modules.

**Consent**

You are invited to take part in a research study involving how bioinformatics and course-based research is taught to college students. The information that you give will be used to help us improve how we teach virtual lab experiences.

This survey is composed of three sections: **(1) identifying information, (2) a post-course assessment, and (3) demographics and STEM identity questions.** Your instructor will have access to Sections (1) and (2), and this may be a component of your grade for the short course. **Section (3) will NOT be shared with your instructors.**

A research team at Tufts University is conducting a research study to determine how the PARE-Seq project can be improved, to evaluate the effectiveness of our curriculum, and to address research questions relating to student experiences with online learning. At the end of these questions, you will be invited to share your answers to help researchers improve this course for future iterations. We need your help to improve how we teach science!

Sharing your answers with Tufts University researchers is voluntary, and **your name will be removed before analysis.** There are no known risks to participating, and there are no personal benefits to you. Refusing to share your answers will involve no penalty or loss of benefits to which you are otherwise entitled. You will authorize your consent to participate at the end of the survey.

People responsible for monitoring this research may also be able to access the data and study records. This includes the Tufts University Institutional Review Board.

**Thank you very much for your help!**

**Section 1: Identifying Information**

Where are you taking this survey?

1. In the United States
2. Outside of the United States

What is your first name? ________

What is your last name? ________

Please select your age range:

1. Under 18
2. 18-24
3. 25-50
4. 50-65
5. 65 or older

Where are you taking this survey?

1. At school during class
2. At school, on my own time
3. In a virtual class, during designated class time
4. Not at school, on my own time
5. Other (Specify) ____

What school or university do you attend?

(Dropdown)

Year of education you are currently completing

1. Senior in high school
2. First year, college
3. Second year, college
4. Third year, college
5. Fourth year, college
6. Other (please specify) ________________________________________________

What is the maximum number of biology classes you have taken?

1. One year in high school
2. More than one year in high school
3. Part of one year in college
4. One year in college
5. More than one year in college

What best describes your major/concentration?

1. Biology
2. Environmental Studies/Science
3. Public Health/Health Sciences
4. Chemistry/Physics
5. Engineering
6. Undecided

Other (please specify) ________________________________________________

**Section 2 (Post-Course Assessment)**

1. What is a metagenome?
   1. Information recovered from one type of bacteria in a sample
   2. **All genetic material recovered from a sample**
   3. All genes present in an environmental sample
   4. DNA recovered from a subset of species in an environmental sample
2. Which statement is TRUE?
   1. Adaptation of populations leads to natural selection
   2. Adaptation of individual organisms leads to natural selection
   3. **Natural selection of individual organisms leads to adaptation of populations**
   4. Natural selection leads to adaptation of an individual organism.
3. What is a disadvantage of using the polymerase chain reaction (PCR) to identify antimicrobial resistance genes (ARGs) for environmental surveillance?
   1. PCR can only copy DNA from one bacterial species at a time
   2. **You can only survey for prespecified targets, and the number of targets you can look for is limited**
   3. PCR amplifies a gene in low abundance, creating millions of copies that we can detect
   4. The short sequences produced by PCR are not long enough to identify resistance genes
4. What is the advantage to sequencing methods that produce long continuous sequences (long “reads”) of thousands of nucleotides compared to methods that produce short sequences (short “reads”) of hundreds of nucleotides?
   1. In order to identify any resistance genes using sequencing, you need long reads
   2. Long-read sequencing of metagenomes is less expensive
   3. **Reads can be long enough to identify both the resistance gene and a sequence that is indicative of the host species**
   4. Long-read sequencing is generally more accurate than short-read sequencing
5. Which ***best*** describes how researchers use metagenomics and bioinformatics to identify genes or families of genes within a very large dataset of DNA?
   1. **A sample of DNA is sequenced and computer programs are used to search these large datasets for nucleic acid sequences that closely match known gene sequences**
   2. A sample of DNA is sequenced and DNA primers that closely match known gene sequences are used to amplify target genes using PCR
   3. A single type of bacteria is isolated, the DNA is extracted, then PCR is used to identify the genes present in bacteria
   4. A sample of DNA is sequenced and computer programs are used to evaluate the quantity of nucleotides present and the error rate of sequencing per gene

**The following passage refers to Questions 7-9.**

You want to analyze different soil samples for the presence of five different antimicrobial resistance genes. When you identify these genes, you also want to know what species of bacteria are hosting these resistance genes. Since there could be millions of different species present in your sample, a single type of resistance gene could be present in several different species of bacteria. Total DNA was extracted from your sample and subjected to long-read DNA sequencing for further analysis.

1. You run a bioinformatics program on the DNA sequences obtained by long-read sequencing to determine if your sequencing data is adequate to proceed with your study. You obtain the outputs below. What information is ***most important***for you to determine whether you should proceed with the analysis?
   1. The total number of reads is less than 50,000
   2. **The median read length is around 6,500 base pairs**
   3. The number of bases sequenced is 32,344
   4. The total number of bases sequenced is over 230,000,000


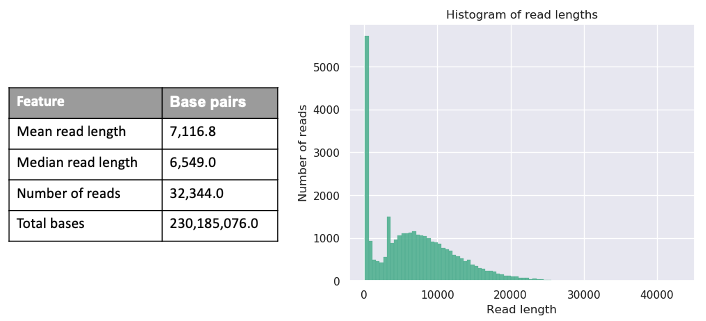


1. In your bioinformatics analysis, the antimicrobial resistance gene alignment tool aligns reads from your sample to a database of tetracycline resistance genes. The analysis yields the table below. What can you conclude from this table?
   1. Tet is the most abundant gene family in your sample
   2. A tetV resistance gene is present in two species in your sample
   3. **9 homologous alignments to resistance genes in the tet family were made, though determining which species they originate from is not possible with this table**
   4. The read identifying tetZ was the longest read produced from conducting long-read sequencing on your sample


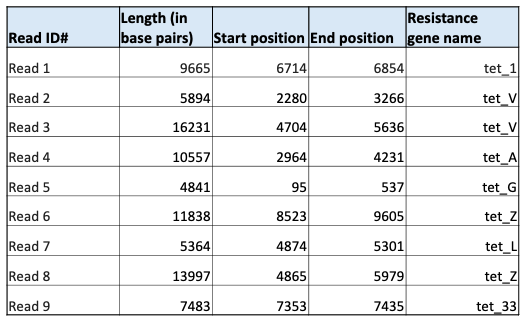


1. Your bioinformatics analysis of the long-read sequence data from your sample yields the antimicrobial resistance gene (ARG)-taxonomy table below, in which reads of a sequence are queried against known antimicrobial resistance gene sequences and also against sequences that characterize the taxonomy of the host species using a homology search algorithm. The same reads are also subjected to homology search to sequences that characterize the taxonomy of the host species. What can be concluded from the sequence data of the soil sample?
   1. The antimicrobial resistance gene, tet_V, is present in one bacterial species in this sample
   2. There is more than one type of tet resistance present in this sample
   3. The gene tet_33 is present in the sample in the species *Tessaracoccus timonensis*
   4. **Both B and C**
   5. All of the above


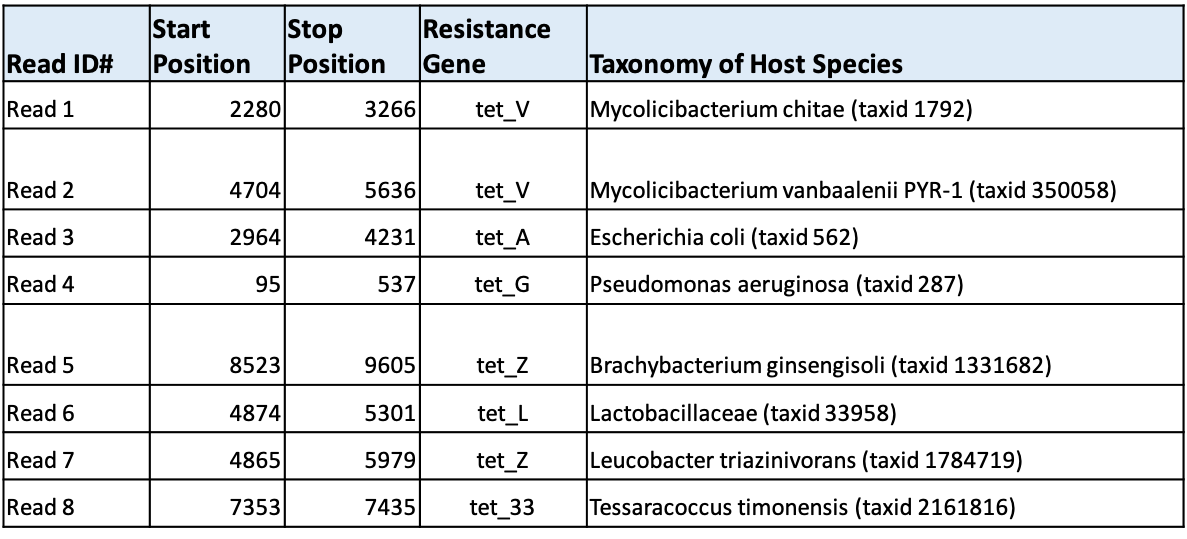


1. (True or False) You quantify antimicrobial resistance genes in two different samples: 1 mL of water and 1g of soil. *You can directly compare the abundance of ARGs between the soil and water sample.*
   1. True
   2. **False**
2. The map below shows the percentage of *E. coli* isolates from blood and cerebrospinal fluid in hospital patients that are resistant to fluroquinolones (a type of antibiotic). What can you determine from the map below?
   1. Which bacteria species are resistant to fluoroquinolones around the world
   2. Where soil and water harbor the most resistance to fluoroquinolones
   3. **Locations worldwide where *E. coli* bacteria have caused infections resistant to fluoroquinolone antibiotics**
   4. The severity of illness caused in patients worldwide by *E.coli* resistant to fluroquinolones


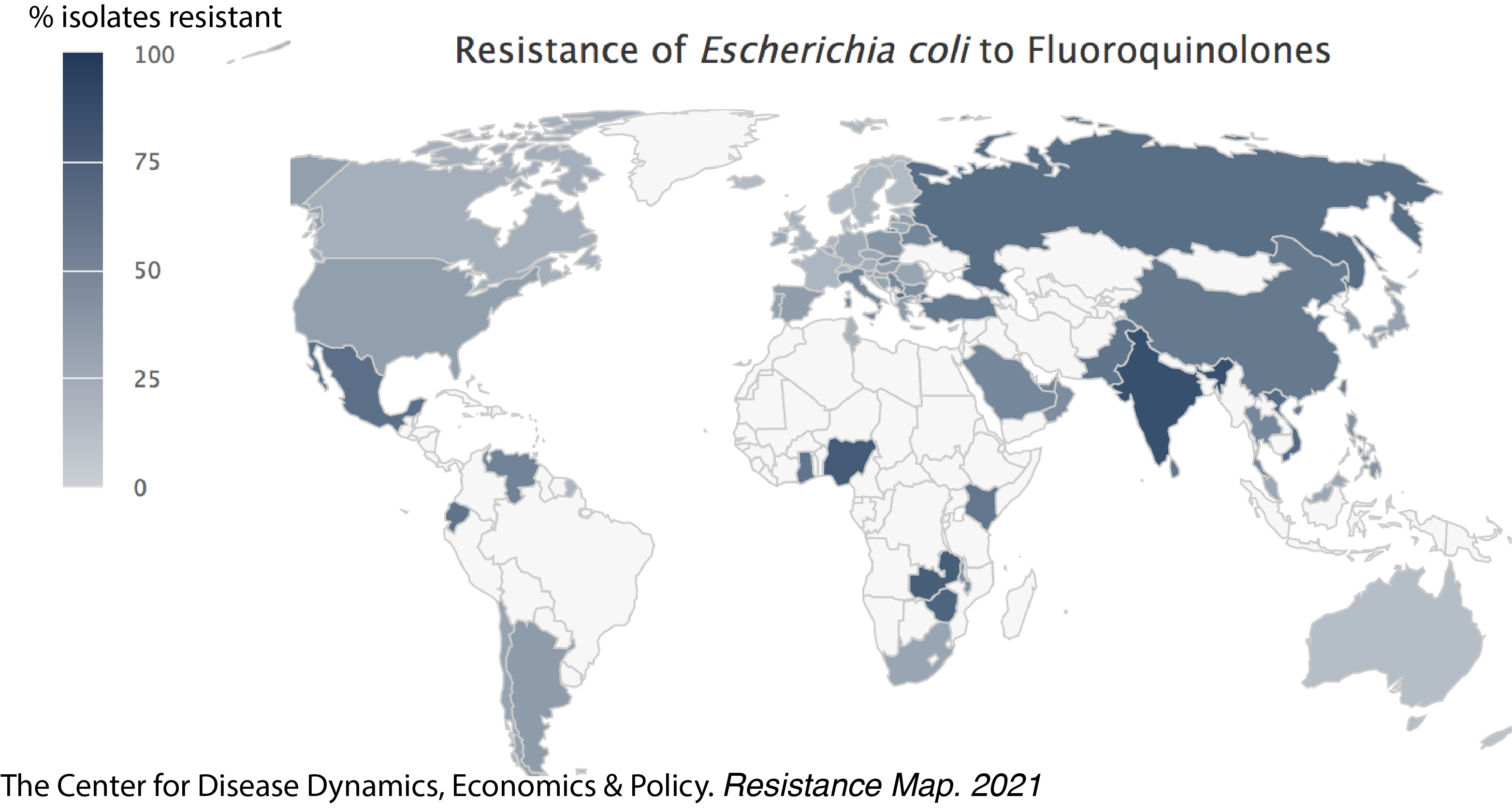


**END GRADED PORTION. PLEASE CONTINUE.**

**Section 3: Sociodemographic and STEM Identity**

Please select the picture that best describes the current overlap of how you see yourself and what your image of a STEM professional is.

**
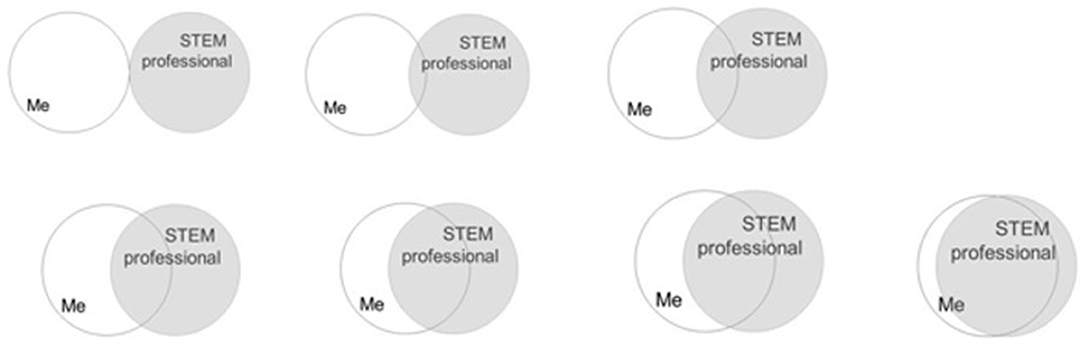
**

I identify as

1. Female
2. Male
3. Non-binary/genderqueer
4. Other:_____
5. Prefer not to answer

Please choose the race and/or ethnicity you most strongly associate with. Select all that apply

1. Hispanic, Latinx, or Spanish origin
2. Black or African-American
3. Asian or Asian-American
4. Bi-Racial or Multi-Racial (2 or more races)
5. Middle Easern or North African
6. Native Hawaiian or Pacific Islander
7. White or European
8. Other (write-in) ______

Have either/any of your parents or guardians received a four-year college degree?

1. Yes
2. No

Are you working a job in addition to taking your courses? If so, how many hours do you work per week?

1. Yes, 1-10 hours
2. Yes, 11-20 hours
3. Yes, 21-30 hours
4. Yes, 31+ hours
5. No

Are you completing this short course on a computer, tablet, or phone?

1. Computer
2. Tablet
3. Phone

Are you the sole user of this computer?

1. Yes
2. No

Please rate the workspace you use to complete your online coursework (1 = many distractions, 6 = very few distractions)

In my class, I completed (select one)

1. Some of the video lectures
2. All of the video lectures
3. All lectures and the Galaxy activity
4. All lectures, the Galaxy activity, and the final project (either lab report or poster)
5. All lectures, the Galaxy activity, a discussion on hypothesis building led by my instructor, and the final project

Overall, *(select from 1 = strongly disagree to 6 = strongly agree)*

1. I enjoyed participating in this short course
2. I think PARE-Seq advanced my understanding of bioinformatics
3. I think PARE-Seq advanced my understanding of metagenomics
4. I think PARE-Seq advanced my understanding of antimicrobial resistance (AMR)

Did you notice diversity in the teaching staff for this short course?

1. Yes
2. No

How impactful is learning from a diverse teaching staff on your interest in the material/STEM? (1 = not at all impactful, 6 = very impactful)

Do you have anything else you’d like to tell us about your experience in PARE-Seq?

[open response]

Overall, I would rate my experience of the PARE-Seq short course

[1-5 stars]

Do you have any comments or feedback for the creators of PARE-Seq (open text response)

Do you authorize Tufts University researchers to view your knowledge questions to improve the quality of the course for future iterations? *Section 3 will NOT be shared with your instructor, and all information will be deidentified before analysis.*

1. Yes
2. No

**Thank you for participating in PARE-Seq and this post-assessment & survey! We appreciate you taking the time to support the improvement of STEM curricula.**
